# Cytokinins on the Move

**DOI:** 10.3389/fpls.2017.00146

**Published:** 2017-02-08

**Authors:** Yolanda Durán-Medina, David Díaz-Ramírez, Nayelli Marsch-Martínez

**Affiliations:** Laboratorio de Identidad Celular de Plantas, Departamento de Biotecnologia y Bioquimica, Centro de Investigación y de Estudios Avanzados del Instituto Politécnico NacionalIrapuato, México

**Keywords:** cytokinin transport, cytokinin uptake, cytokinin export, ENT, PUP, ABCG, cytokinin signaling

## Abstract

Cytokinins are important signals that participate in different plant processes, and are well known for their strong influence in plant development. With the years, knowledge has been built about their effects, chemical nature, metabolism, and signaling mechanisms. However, one aspect about cytokinins that has been lagging behind is cytokinin transport. Recent reports are providing more information about how cytokinins are transported and how their transport is connected to their effects in development. This review provides a general overview of what is known about cytokinin transport, with a focus on the latest reports.

For a long time, the molecular mechanisms of cytokinin action were poorly understood, because information about the physiological role of cytokinins came from external application experiments, and did not involve the use of plants with altered levels of or responses to cytokinin. It took over 40 years to identify the first genes involved in cytokinin signaling. In 1996, using an activation tagging strategy, Kakimoto isolated *Arabidopsis thaliana* mutants exhibiting cytokinin responses that were independent of cytokinin application. In this screen, *CYTOKININ INDEPENDENT 1* (*CKI1)* was identified (Kakimoto, 1996). Though unlikely a cytokinin receptor (Yamada, 2001), *CKI1*, encoding an integral membrane protein similar to bacterial two-component His kinases, provided the first hint about cytokinin perception and signaling. The first authentic cytokinin receptor, CYTOKININ RESPONSE 1 (CRE1), was identified by Inoue et al. (2001) by screening for *Arabidopsis* mutants whose hypocotyl explants failed to form shoots in medium with high cytokinin/auxin ratio, and it was found to sense cytokinins in heterologous systems (Inoue et al., 2001; Suzuki et al., 2001; reviewed in Heyl et al., 2012).

Further research on cytokinin receptors yielded insights into the biochemical properties and functions of these sensor histidine kinases. Their nature suggested that other downstream elements of a two-component signaling pathway (TCS) could participate in the signal transduction, such as *Arabidopsis* histidine-containing phosphotransfer proteins (AHPs) and *Arabidopsis* response regulators (ARRs) (summarized in Kieber and Schaller, 2010; Hwang et al., 2012).

Cytokinin-overproducing plants obtained by activation tagging helped to identify an endogenous plant isopentenyl transferase (IPT) that produced active CKs *in planta* (Zubko et al., 2002). Moreover, additional knowledge about the biological function of cytokinins was obtained from cytokinin-deficient plants, such as transgenic tobacco plants overexpressing genes that encode catabolic cytokinin oxidase (CKX) enzymes from *Arabidopsis* (Werner et al., 2003).

Further genetic and molecular analyses boosted significant progress in unraveling cytokinin metabolism, perception, and signal transduction (summarized in the following reviews: Sakakibara, 2006; Hirose et al., 2008; Del Bianco et al., 2013; El-Showk et al., 2013; Kieber and Schaller, 2014; Zürcher and Müller, 2016).

However, less knowledge was available about the molecular mechanisms involved in cytokinin transport. Mainly, three kinds of cytokinin transporters have been reported to date: purine permeases (PUP), equilibrative nucleoside transporters (ENT), and G subfamily ATP-binding cassette (ABCG) transporters (reviewed in Kudo et al., 2010; Girke et al., 2014; Borghi et al., 2015; summarized in **Table 1**).

**Table 1:** Cytokinin transporter families.

Transporter family	Evidence of cytokinin transport activity	Members with experimental evidence for cytokinin transport	Assayed cytokinin^∗^, K (when available), system	Expression pattern	Subcellular localization	Reference
**PUP**	Cytokinin uptake (zeatin, kinetin, isopentenyladenine benzyladenine, and some ribosides)	AtPUP1	*K*_i_ = 20–35 μM (kinetin and zeatin vs. adenine transport), *K*_m_ = 40 μM (*trans-*zeatin), in yeast	Leaves, stems, flowers, siliques, hydathodes, stigmata		Gillissen et al., 2000; Bürkle et al., 2003	**Review:** Girke et al., 2014
		AtPUP2	Adenine transport inhibition by iPA, *trans* and *cis*-zeatin, kinetin, BAP and tZR, in yeast	Flowers, roots, leaf vasculature (phloem), pollen		Bürkle et al., 2003	
		AtPUP14	*trans*-zeatin, iPA, BA, in plant-derived protoplasts and microsomes	Many organs and stages	Plasma membrane	Zürcher et al., 2016	
		OsPUP7	Higher iP and iPR levels in the mutant plant	Stigma, style, stamens, hull vein, vascular bundle of roots, culms and leaves		Qi and Xiong, 2013	

**ENT**	Cytokinin uptake (ribosides, inactive transport forms)	AtENT8 AtENT3	Reduced uptake efficiency of iPR, in mutant hypocotyls	*ENT8*: SAM, hypocotyl, petioles, stems, flowers, siliques		Sun et al., 2005	**Review:** Girke et al., 2014
		AtENT6	*K*_m_ = 17 μM iPR, *K*_m_ = 630 μM tZR, in yeast	Root, leaf, and flower vasculature, and stomata	Plasma membrane	Wormit et al., 2004; Hirose et al., 2008	
		OsENT2	*K*_m_ = 32 μM iPR, *K*_m_ = 660 μM tZR, in yeast	Vasculature (grains, roots, stems, and leaves), and roots (stele, lateral roots)		Hirose et al., 2005	

**ABCG**	Cytokinin export (*trans-*zeatin type)	AtABCG14	Reduced *trans-zeatin* root-to-shoot translocation in mutant plants	Mainly roots (phloem, procambium and pericycle)	Plasma membrane	Ko et al., 2014; Zhang et al., 2014 Reviews: Borghi et al., 2015; Hwang et al., 2016


*^∗^More biochemical data for transport of other molecules, such as adenine, is available for these transporters, but this table only contains data related to cytokinin transport. iPA or iP, isopentenyladenine; iPR, isopentenyladenosine; BAP or BA, benzylaminopurine or benzyladenine; tZR, *trans-*zeatin riboside.*

The first PUP transporter was identified through a complementation assay using an adenine uptake deficient yeast mutant transformed with an *Arabidopsis* cDNA library. Competition assays indicated that, besides adenine, PUP1 could also transport the cytokinins kinetin and zeatin, and at a lower extent their ribosides (Gillissen et al., 2000). In this way, a new membrane protein family of 21 members was identified (Schwacke et al., 2003). Further analyses of the transport function of two other members of the family, PUP2 and PUP3, showed that PUP2 could also transport cytokinins (*trans*- and *cis*-zeatin, kinetin, benzyladenine, isopentenyladenine, and to a lesser extent *trans*-zeatin riboside), while no transport evidence could be obtained for PUP3 (Bürkle et al., 2003). Moreover *PUP1. PUP2* and *PUP3* presented very different expression patterns (in hydathodes and stigmatic papillae, vasculature and pollen, respectively), giving a hint about their physiological roles in the plant (Bürkle et al., 2003). Later studies on cytokinin uptake kinetics in *Arabidopsis* cell cultures and seedlings, suggested the existence of low and high-affinity transport systems. Furthermore, an exploration of expression data of more than 12 *PUP* genes revealed that they were expressed in diverse tissues and levels (Cedzich et al., 2008). Hildreth et al. (2011) have proposed that PUPs are only present in vascular plants, where they can transport also other molecules (besides cytokinins) such as caffeine and nicotine (Gillissen et al., 2000; Bürkle et al., 2003; Hildreth et al., 2011; Jelesko, 2012; Qi and Xiong, 2013; reviewed in Girke et al., 2014).

More evidence for PUP function in cytokinin transport has been obtained from studies in rice. Qi and Xiong (2013) identified 12 *OsPUP* members, which also present varied expression patterns in different tissues and developmental stages. They characterized *OsPUP7*, exploring its expression and making use of an available insertional mutant (Qi and Xiong, 2013). *OsPUP7* was found to be expressed mainly in the pistils, stamens, and the vasculature of different organs, and to be induced by the addition of abscisic acid and kinetin. Because isopentenyladenine and isopentenyladenosine levels were altered in the mutant, the authors proposed that OsPUP7 is also able to transport cytokinins and derivatives. However, levels of *trans-*zeatin and *trans-*zeatin riboside were not affected, leading the authors to suggest that this transporter has a selective transport activity. The insertional mutant presented clear alterations in organ size and flowering time, and had increased sensitivity to abiotic stresses (osmotic and saline stress) and hormone treatments (abscisic acid and benzyladenine). However, how the function of these transporters was affecting the cytokinin signaling response of cells during plant development was unknown.

Recently, a report by Zürcher et al. (2016) shed light on these questions. To identify regulatory steps in the cytokinin signaling response pathway, they used the *Arabidopsis* embryo at the heart stage as a model. When embryos were treated with the cytokinin benzyladenine, the signal of the cytokinin-sensitive two-component sensor *TCSn:GFP* (Zürcher et al., 2013) expanded from the provascular tissue to other tissues. However, no response was detected in the prospective cotyledons. The authors noticed that the lack of *TCSn:GFP* response in the prospective cotyledons was most likely not due to a lack of cytokinin receptors or downstream signaling elements. The *Arabidopsis* histidine kinase 4 (*AHK4/CRE1)* cytokinin receptor is expressed there. Moreover, downstream signaling appeared to be functional as overexpression of the cytokinin-independent constitutively active *CKI1* kinase, which acts upstream of the AHPs (Deng et al., 2010), caused ubiquitous *TCSn:GFP* activation. Therefore, the authors hypothesized that cytokinin transporters could be responsible for the lack of *TCSn:GFP* activation in the prospective cotyledons. To test their hypothesis, they surveyed the expression patterns of *PUP* transporter genes to find one expressed in this tissue. Among them, they found *PUP14* to be highly expressed in many stages and tissues, including the prospective cotyledons suggesting that it could be negatively affecting the cytokinin response in this tissue. Indeed, induction of *PUP14* silencing (using an artificial microRNA, *amirPUP14)* caused ectopic cytokinin signaling in the prospective cotyledons, indicating that PUP14 could restrict cytokinin signaling. Later in development, these tissues with ectopic signaling presented morphological defects (also observed in the nascent root meristem). Furthermore, constitutive *PUP14* ectopic expression had deleterious effects, and its induction caused a reduction in cytokinin signaling. Uptake experiments using labeled *trans-*zeatin confirmed that PUP14, which was localized at the plasma membrane, could transport this cytokinin. Moreover, adenine and bioactive cytokinins (isopentenyladenine and benzyladenine) could inhibit *trans*-zeatin transport in competition assays. In contrast, neither the transport form *trans-*zeatin riboside, auxin, nor allantoin, inhibited uptake by PUP14. The authors reasoned that uptake of bioactive cytokinins by PUP14, together with the effects of PUP14 down or up-regulation in the expression *TCSn:GFP* reporter line, could suggest that this transporter could be depleting cytokinins from the apoplast, making them unavailable to be sensed by plasma membrane-localized receptors. To test this, they compared the effect of PUP14 uptake, to the effects of CKXs (cytokinin-degrading enzymes) targeted for secretion to the apoplast or localized in the cytoplasm, in the cytokinin signaling response. They found that both enzymatic clearing of external cytokinins, and cytokinin uptake had a similar effect on the cytokinin signaling response, suggesting that apoplastic and not cytoplasmic cytokinins initiate signaling as reported by the *TCSn:GFP* line.

Therefore, the authors proposed a model in which PUP14-mediated uptake of cytokinins depletes them from the apoplast, constraining, and therefore contributing to define the topography of cytokinin signaling responses that guide morphogenesis in a developing plant (the model proposed is depicted in **Figure 1**).

**FIGURE 1:**
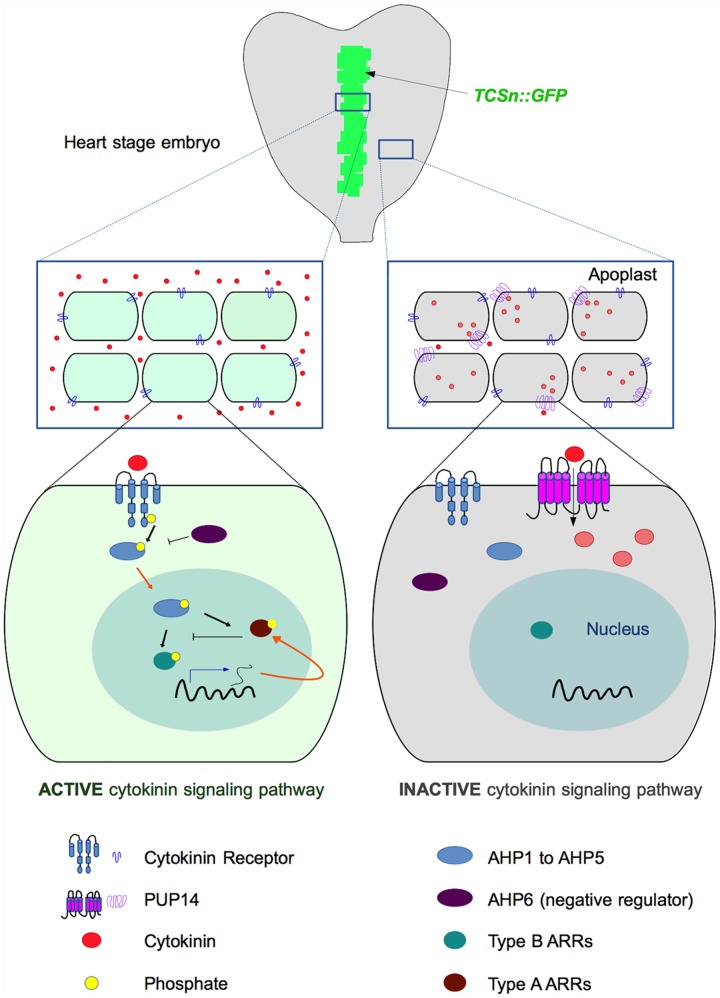
**Proposed mechanism of cytokinin-signaling restriction by PUP14 (based on models published by Bürkle et al., 2003; Zürcher et al., 2016).** Top: Diagram depicting the region of cytokinin response (indicated in green) revealed by the *TCSn:GFP* marker in a heart-stage embryo. Zürcher et al. (2016) proposed that PUP14 import of apoplastic cytokinins to the cytosol causes their depletion from the apoplast and makes them unavailable for the membrane-bound AHK receptors that initiate the signaling cascade that activates the TCS response. In the middle and bottom diagrams, cytokinin responsive cells are light green, while non-responsive cells are represented in gray. In the responsive cells (left), cytokinins are perceived by the AHK receptors, which autophosphorylate and subsequently transfer the phosphate (black arrows represent phosphorylation) to *Arabidopsis* histidine-containing phosphotransfer proteins (AHP1 to AHP5). Then, the AHPs enter the nucleus (orange arrows represent intracellular traffic) and phosphorylate type B *Arabidopsis* response regulators (ARRs), which activate transcription (blue arrow) of CK response genes, reported by the *TCSn:GFP* marker line and including type A ARRs (negative regulators of cytokinin signaling, as indicated by blocked lines). In the right part of the diagram, the expression of PUP14 promotes the depletion of cytokinins from the apoplast and the cytokinin response (reported by the *TCSn:GFP* marker) is not activated, even when exogenous cytokinins are applied. Cytokinins are represented by red circles, and internalized cytokinins are represented by light red circles. For simplicity, only one TCS element or PUP14 transporter per cell is depicted.

With the proposed model, new questions emerge. For example, it would be interesting to assess the ratio of transporters vs. receptors in the cell, or at least the tissue, under study to provide a better understanding of their competing interactions with cytokinins, especially considering that high affinities have been reported for the cytokinin receptors (in the low nM range, Yamada, 2001; Romanov et al., 2005, 2006; Lomin et al., 2015, reviewed in Heyl et al., 2012). Affinities for cytokinins have been measured for only two PUPs (PUP1 and 2, *K*-values in the μM range, Gillissen et al., 2000; Bürkle et al., 2003) using different methods, and they do not seem to be comparable to the reported receptor affinities. Therefore, to better understand the physiological role of PUP14 and further support the proposed model, it would be very informative to measure its affinity for cytokinins and compare it to the affinities of the receptors under the same conditions. In this comparison, conditions such as pH should be considered. In the case of cytokinin receptors, changes in their sensitivity depending on pH have been reported (Romanov et al., 2006; Lomin et al., 2015), and this is a relevant factor to consider, since different cellular and extracellular compartments have different pHs (Lomax et al., 1985; Li et al., 2005). Previous studies have reported cytokinin receptor localization, and cytokinin sensing, mostly at the endoplasmic reticulum (Caesar et al., 2011; Lomin et al., 2011; Wulfetange et al., 2011). In this regard, we are still missing key information that can reconcile the data that indicates that cytokinins are sensed at the plasma membrane, as in the proposed model for PUP14 function, with the data that indicates that cytokinins are sensed at the endoplasmic reticulum, as reported in the works cited above. Here, further research in cytokinin transport may also contribute to unravel this enigma, answering questions regarding how cytokinins reach each of these locations inside and outside the cell, and the advantages of a sophisticated system of cytokinin depletion over the downregulation of receptors in a group of cells.

The second family of cytokinin transporters, the ENTs, were found initially as putative plant nucleoside transporters with similarity to the human transporters *hENT1* and *hENT2*, and reported to localize at the plasma membrane (Li and Wang, 2000). Complementation of a yeast mutant unable to synthesize adenine, with *Arabidopsis* cDNA, demonstrated that *AtENT1* could transport adenosine (Möhlmann et al., 2001; reviewed in Girke et al., 2014), while its silencing affected pollen germination (Bernard et al., 2011). FURTHER characterization of the nucleoside transport capabilities and expression of other *AtENTs* followed (Li et al., 2003; Wormit et al., 2004; Traub et al., 2007). The finding of an *AtENT8* mutation as a suppressor of the altered phenotypes of a cytokinin overproducing plant, together with the characterization of AtENT6 and the rice OsENT2 in yeast, provided evidence of a role for ENTs in cytokinin transport (Hirose et al., 2005, 2008; Sun et al., 2005). For *AtENT3*, a loss-of-function mutant presented reduced cytokinin uptake (Sun et al., 2005), but cytokinin transport activity was not detected when the transporter was expressed in yeast (Hirose et al., 2008). Until now, the data indicates that the cytokinin species transported by a subset of the ENTs are the riboside forms of isopentenyladenine and *trans-*zeatin (isopentenyladenine riboside and *trans-*zeatin riboside) (Hirose et al., 2005, 2008; Sun et al., 2005; reviewed in Girke et al., 2014). These forms are not active cytokinins, and are considered to be transport forms (reviewed in Hirose et al., 2008; Lomin et al., 2015 reported *in planta* binding assays to receptors showing strongly reduced affinity of riboside forms). Also, as reported for PUPs, ENTs can transport many non-cytokinin molecules, opening the question of whether only some members are involved in cytokinin transport, and whether they may play broader roles, not specific to cytokinin homeostasis.

Current evidence indicates that both PUP and ENT transporters participate in cytokinin uptake. But are there transporters that have been shown to export cytokinins?

Different cytokinin species are translocated between different plant organs. *trans*-zeatin types are transported via the xylem from roots to shoots and isopentenyladenine types via the phloem systemically or from shoots to roots (reviewed in Kudo et al., 2010; Zürcher and Müller, 2016). Therefore, the existence of cytokinin exporters was suspected, but remained an enigma until 2014. Then, two groups independently reported that the *Arabidopsis* ABCG14 transporter functions as a cytokinin exporter that loads cytokinin in the root xylem (Ko et al., 2014; Zhang et al., 2014). In one case, using a genetic approach to characterize the ABCG proteins, Zhang et al. (2014) found that knock-out mutations in *ABCG14* led to defects in the long-distance translocation and distribution of the root-synthesized *trans-*zeatin type cytokinins, evidenced by their overaccumulation in the root and reduced content in the shoot. These defects caused morphological alterations such as small inflorescences and rosettes, slender stems with altered vasculature, and short primary roots. Efflux pump activity was demonstrated by employing radiolabeled *trans-*zeatin species in *in planta* feeding and detached leaf assays (Zhang et al., 2014). In the other case, to elucidate the molecular mechanism underlying the translocation of cytokinins, Ko et al. (2014) searched for genes that were highly expressed in root phloem companion cells, and co-expressed with the cytokinin synthesis *adenylate isopentenyltransferase 3* (*IPT3*) gene and with genes induced by cytokinin treatments. In this way they found *ABCG14*, and confirmed that this gene was also induced in cytokinin-treated seedlings. The developmental defects observed in the *abcg14* insertional knock-out mutant resembled those of cytokinin biosynthetic mutants, and were rescued with exogenous *trans-*zeatin, but not with isopentenyladenine, applications. As Ko et al. (2014) and Zhang et al. (2014) also tested transport capabilities and observed increased accumulation of *trans-*zeatin type cytokinins in the root and reduced content in the shoot. Moreover, they also found that *ABCG14* is expressed in the vasculature, and that the protein it encodes is localized at the plasma membrane. Interestingly, like the application of exogenous *trans-*zeatin, the grafting of *abcg14* shoots to wild type roots could rescue defective shoot growth, suggesting that the translocation of cytokinins from roots to shoots, mediated by ABCG14, is necessary for proper shoot growth and development (Ko et al., 2014).

The ABCGs belong to a subfamily of ATP-binding cassette (ABC) transporters that participate in various biological processes, including the transport of other hormones (reviewed by Borghi et al., 2015). Interestingly, plants have many more ABC transporters than other organisms, and ABCG transporters are particularly abundant in plants (reviewed by Hwang et al., 2016). These transporters have been clustered in six groups (Rajsz et al., 2016), and although the activity of many has been characterized, the biological and biochemical functions of many ABCG proteins remain undetermined and it is possible that other proteins of this subfamily can also export cytokinin (reviewed in Borghi et al., 2015; Hwang et al., 2016).

In summary, the work on PUP, ENT and ABCG transporters has provided more pieces to put together the puzzle of cytokinin transport and to improve the current understanding of cytokinin action.

Still, there are many questions that remain open about these matters. For cytokinin transport, some of the many possible questions that come to mind are: Which roles do the different members of these families play in the local, paracrine and systemic effects of cytokinin? Do other members of each transporter family have the same biochemical functions as the members that have been studied? For example, human ENTs can facilitate nucleoside transport in a bidirectional fashion, and some have been associated with nuclear or endoplasmic reticulum membranes (reviewed in Young et al., 2008). Do members of this family in plants function or localize in similar ways? Moreover, phloem cytokinin (isopentenyladenine type) transport from shoot to root has an important function in root vascular development and nodulation control (Bishopp et al., 2011; Sasaki et al., 2014; brief summary: Muraro et al., 2011). Do members of one or more of these families participate in this translocation? Are there more cytokinin transporters that remain to be discovered?

It will be very interesting to further explore the localization, transport capabilities and role of all the members of these transporter families in cytokinin movement, their impact on plant development and other processes, and integrate the new knowledge in the current models of cytokinin homeostasis and action.

## Author Contributions

All the authors revised published information, discussed and prepared the table, and drafted the manuscript. DD-R and YD-M prepared the figure, and NM-M prepared the table and edited the final versions of the manuscript.

## Conflict of Interest Statement

The authors declare that the research was conducted in the absence of any commercial or financial relationships that could be construed as a potential conflict of interest.
